# Evaluation of Anxiolytic-Like Effect of Aqueous Extract of *Asparagus* Stem in Mice

**DOI:** 10.1155/2013/587260

**Published:** 2013-11-20

**Authors:** Long Cheng, Guo-feng Pan, Xiao-bo Sun, Yun-xiang Huang, You-shun Peng, Lin-yan Zhou

**Affiliations:** ^1^Institute of Medicinal Plant Development, Chinese Academy of Medical Sciences, Beijing 100094, China; ^2^Department of TCM, Beijing Shijitan Hospital Affiliated to Capital Medical University, Beijing 100038, China; ^3^Asparagus Engineering Research Centers of Hebei Province, Qinhuangdao 066008, China; ^4^Hebei Normal University of Science & Technology, Qinhuangdao 066004, China; ^5^Institute of Agro-Products Processing Science & Technology, Chinese Academy of Agricultural Sciences, Beijing 100193, China

## Abstract

There are few studies on the neuropharmacological properties of asparagus, which was applied in Chinese traditional medicine as a tonic and heat-clearing agent. The present study was designed to investigate the anxiolytic-like activity of the aqueous extract of asparagus stem (AEAS) using elevated plus maze (EPM) and Vogel conflict tests (VCT) in mice. AEAS significantly increased the percentage of time spent in open arms in EPM, when compared with control group. In the Vogel conflict drinking test, the numbers of punished licks increased to 177% and 174% by the treatment of AEAS at the doses of 1.5 and 3.0 g/kg (250 and 500 mg sarsasapogenin per kilogram of body weight), compared with control group. The serum cortisol level decreased significantly, at the same time. In conclusion, these findings indicated that the aqueous extract of asparagus stem exhibited a strong anxiolytic-like effect at dose of 1.5 and 3.0 g/kg (250 and 500 mg sarsasapogenin per kilogram of body weight) in experimental models of anxiety and may be considered an alternative approach for the management of anxiety disorder.

## 1. Introduction

Anxiety and related disorders, most common mental illnesses worldwide, represent a prominent healthcare problem [1–4]. Reports from the World Health Organization (WHO) suggest that anxiety and related disorders will become the second leading cause of disability in both developed and developing countries by the year 2020 [5]. Benzodiazepines are among the first line of drugs that have been extensively used to treat several forms of anxiety [6]. Although benzodiazepines have well-known benefits, their side effects are prominent [7]. Therefore, the search for new therapeutic agents continues. Interest in alternative medicine and plant-derived functional food to conquer stress and promote relaxation has increased recently. For example, the concept of a relaxation drink first emerged in Japan in 2005 [8].

*Asparagus officinalis L.*, a well-known nutritious and healthy vegetable, was applied widely in the traditional Chinese medicine clinic practice, as a tonic, heat-clearing, antitussive, and diuretic agent. In recent years, various healthful effects of the asparagus have been scientifically verified [9–13]. Asparagus industrial product development is in the ascendant, in China. In early 2013, the Chinese government authorized the aqueous extract of asparagus stem (AEAS) as a natural functional food and beverage ingredient. The present study investigated the potential anxiolytic effect of AEAS in mice. 

## 2. Materials and Methods

### 2.1. Plant Material

Material was collected from cultivation in Hangu administration zone of Tangshan, a vegetation zone that belongs to Qinhuangdao Changsheng Agricultural Technology Development Co., Ltd. Botanical samples were identified by Professor Peng of Hebei Normal University of Science and Technology, Qinhuangdao, China.

### 2.2. Extract Preparation

Aqueous extract from the stem of *Asparagus officinalis L*. was provided by Qinhuangdao Changsheng Agricultural Technology Development Co., Ltd., Hebei Province, China. To obtain the aqueous extract from the woody stem of asparagus, fresh plant material (60.0 kg) was cleaned, crushed, and then extracted with drinking water (plant/water ratio 1 : 8 w/v; water temperature: 85 ± 5°C) by dynamic maceration for 5 h. After filtering, the solution of asparagus stem was treated with spray drying to dryness, yielding extract powder (1.0 kg AEAS).

### 2.3. Extract Character

**Figure 1:**
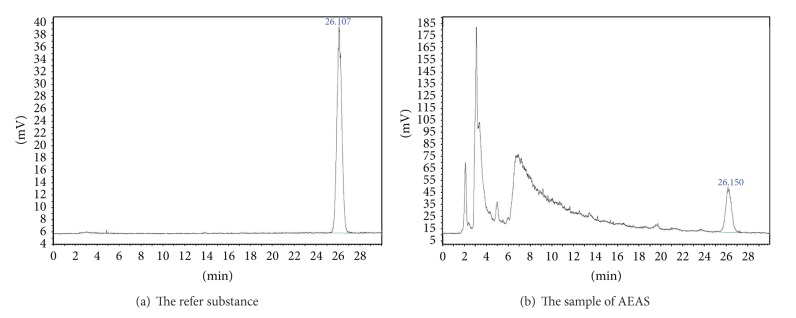
The chromatography of sarsasapogenin from AEAS.

**Figure 2:**
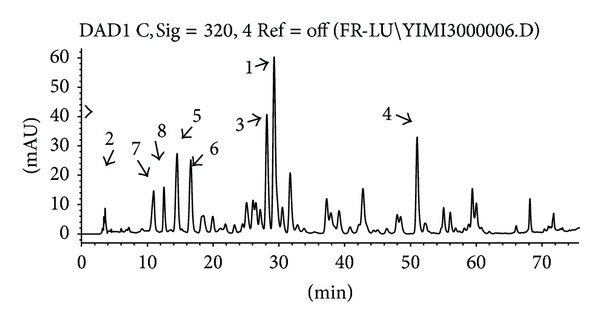
The HPLC-UV fingerprint of the flavonoids from AEAS.

The nutrient and chemical components of this studied extract were identified and quantified (shown in Table 1). Amino acid composition analysis was performed by a fully automatic amino acid analyzer (Hitachi L-8900, Japan). The data were shown in Table 2. The main biologically active constituents of asparagus are the steroidal saponins and flavonoids. For quantitative determination of the steroidal saponins, the AEAS was refluxed with hydrochloric acid solution. The sarsasapogenin from asparagus was determined by reversed phase high performance liquid chromatographic method with evaporative light scattering detection (RP-HPLC-ELSD) (shown in Figure 1). The content of sarsasapogenin was 17.80%. The fingerprint of the flavonoids from AEAS was established by HPLC-UV (shown in Figure 2 and Table 3). Asparagus extract solution was mixed with diethyl ether then stirred. The diethyl ether layer was separated and evaporated to dryness. The resulting residue was dissolved in methanol as sample solution for fingerprint analyses. 

The analyses were performed using a Zorbax C_18_ column and a diode array detector in an Agilent1100 HPLC system. The mobile phase was 0.5% formic acid solution (solvent A) and methanol (solvent B), at a flow rate of 0.7 mL/min. The gradient elution was 0–20 min, 80%–70% A, 20%–30% B, 20–45 min, 70%–60% A, 30%–40% B, 45–60 min, 60%–50% A, 40%–50% B, 60–70 min, 50%–30% A, 50%–70% B, and 70–75 min, 30%–0% A, 70%–100% B. The UV detector wavelength was set at 320 nm. Five compounds were identified by the spectral feature of UV, MS, and MS/MS, (Table 3). The main 8 peaks were quantified by comparing their peak areas with the refer substance (Peak 1). 

### 2.4. Drug

Distilled drinking water was used as vehicle. Diazepam (DZP) was used as the reference drug for anxiolytic activities. 

### 2.5. Animals

Male Imprinting Control Region mice (CD-1, ICR, 18~22 g) were purchased from Beijing Vital River Laboratories Co., Ltd. (Beijing, China). Animals were housed in a controlled environment (temperature 23° ± 2°C, relative humidity 50% ± 10%, 12 h light/dark cycle) and allowed free access to standard diet. They were acclimatized for 5 days prior to the initiation of the test. 

For the repeated dose toxicity study, Sprague Dawley (SD) rats were used. The rats were housed in stainless steel cages, kept under controlled conditions of temperature (23 ± 2°C), relative humidity (55 ± 10%) and ventilation (more than 10 times/hour) with a 12-hour light/dark cycle, and allowed free access to food and water throughout both the acclimation and experimentation periods. All animals were maintained in accordance with the Beijing Laboratory Animal Management Regulations.

### 2.6. Elevated Plus Maze

Evaluation of the possible anxiolytic effect was performed according to the previous literature [14–16]. Shanghai Manpu Biotechnology Co., Ltd., furnished the EPM apparatus (mode: RD1108-EPM-M), which comprised two open (30 cm × 5 cm) and two closed arms (30 cm × 5 cm × 15 cm) that extended from a common central platform (5 cm × 5 cm). To prevent mice from falling out of the maze, a ledge (2 mm H) surrounded the open arms. The floor and the walls of each arm were painted black. The entire maze was elevated 60 cm above floor.

Experiments were conducted in a dimly lit, quiet room. We pretreated male mice orally with vehicle or different doses (0.6, 1.5, or 3.0 g/kg) of AEAS (equivalent to 100, 250, and 500 mg sarsasapogenin per kilogram of body weight) 30 min before placing them on the EPM. One group of animals received diazepam (3 mg/kg, p.o.) as reference drug. To begin a test session, mice were placed in the center of the maze facing one of the open arms and allowed to walk freely for 5 min. After each trial, the maze was wiped clean with a damp sponge and dried with paper towels. All the tests were recorded by video camera.

Entry into an arm was defined as the animal placing all four paws over the line marking that area. Standard spatiotemporal measures were scored: the number of entries in the open and the closed arms, the total number of arm entries, and the time spent in the different parts of the maze (open and closed arms). The ratio of open arm entries and closed arm entries to the total number of arm entries was also calculated during a 5 min test period for each animal.

### 2.7. Vogel Conflict Test

Mice were habituated for 5 days and then divided randomly into five groups. Control group animals received drinking water only. DZP group received 3.0 mg/kg of diazepam (p.o.). AEAS groups received 0.6, 1.5, or 3.0 g/kg AEAS (equivalent to 100, 250, and 500 mg sarsasapogenin per kilogram of body weight) per day, respectively. This procedure continued for 7 days. At 8:30 am of day 8, rats were treated with AEAS, DZP, or drinking water. The Vogel conflict test was performed about 30 min later.

 The Vogel conflict test apparatus (mode: AES-340, AniLab Software and Instruments Co., Ltd.) was applied, essentially using the procedure originally described by Vogel et al. [17]. The VCT was performed in a Plexiglas box with a stainless steel grid floor. The metallic spout of a drinking bottle that contained water projected into the box. The simultaneous contact of the animal with the spout and the grid floor closed an electrical circuit controlled by a sensor, producing seven pulses of water per second whenever the animal was in contact with both components. Each pulse was considered a lick. After every 20 licks, the animal received a 0.5 mA footshock for 2 s. The sensor recorded the total number of licks and shocks delivered during the test period. The entire apparatus was located inside a sound-attenuated cage. After completing the test session, all rats were anesthetized. The femoral arterial blood was collected and centrifuged to get serum sample. Their brains were rapidly dissected (not more than 3 min) on ice, then hippocampus were dissected, weighed, and homogenized in 0.1 M phosphate buffer solution by manual homogeniser, centrifuged at 10,000 rpm for 20 min to remove supernatant and stored at −20°C until analysis.

### 2.8. Neurochemical Assay

The cortical levels in serum were determined by commercially available radioimmunoassay kits (Beijing North Institute of Biological Technology Co., Ltd., Beijing, China). Catecholamine (epinephrine, norepinephrine, and dopamine) and 5-hydroxytryptamine (5-HT) levels in serum and tissue homogenate were determined by enzyme immunoassay kits (Cusabio Biological Technology Co., Ltd., Wuhan, China).

### 2.9. Repeated Doses Toxicity

For evaluation of the edible safety, 13 weeks repeated dose oral toxicity study was performed in rats. SD rats were assigned into four groups randomly: Group I: control group; Group II: treated with AEAS 1.25 g/kg (equivalent to 208 mg sarsasapogenin per kilogram of body weight); Group III: treated with AEAS 2.50 g/kg (equivalent to 417 mg sarsasapogenin per kilogram of body weight); Group IV: treated with AEAS 5.00 g/kg (equivalent to 835 mg sarsasapogenin per kilogram of body weight). The doses were designed according to the previous acute study result and the recommended daily dose (equivalent to 25-, 50-, and 100-fold of recommended dose, 3.0 g/d for adult). 

Rat gross behavior, mortality, body weight gain, and food intake were recorded throughout the repeated treatment. After 13 weeks feeding, all rats were deprived of food (but not water) overnight and then were anesthetized. The urine and blood sample were collected then detected, respectively. The organs (liver, kidneys, adrenal glands, spleen, lungs, heart, brain, and so forth) were harvested, weighed, and then processed for histopathological examination. Hematological examination was carried out by an automatic analyzer (Hitachi 7600-010, Hitachi Co., Hyogo, Japan). The urine analyses were performed with urinalysis strips (Suzhu pharmaceutical Co., Ltd., Jiangsu, China).

### 2.10. Statistical Analysis

Data were processed to give group mean values and standard deviations. The quantitative data were assessed by one-way analysis of variance (ANOVA). If the *F* distribution was significant, a *t*-test was used to specify differences between groups. *P* < 0.05 was considered statistically significant. The SPSS 11.0 software package (SPSS Inc., Chicago, IL, USA) was used for the statistical test.

## 3. Results

### 3.1. Effect of AEAS in EPM Test

**Figure 3:**
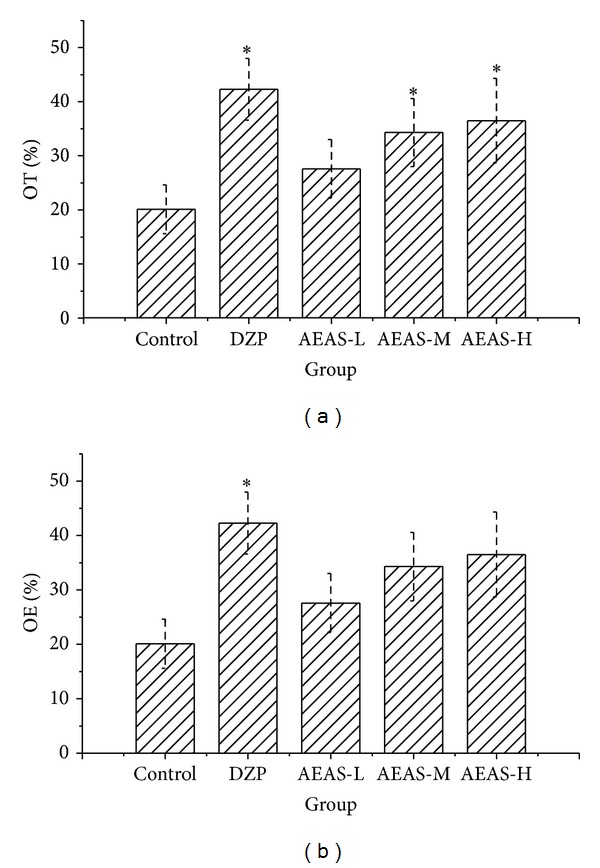
The effect of AEAS on the EPM test in mice ((b) for OE% and (a) for OT%). The aqueous extract of asparagus stem (AEAS), elevated plus maze (EPM), and diazepam (DZP). The percent of open arm entries to the total number of arm entries (OE). The percent of time spent in the open arm to the total time in the open and close arms (OT). The dose of aqueous extract of asparagus stem 0.6 g/kg (AEAS-L). The dose of aqueous extract of asparagus stem 1.5 g/kg (AEAS-M). The dose of aqueous extract of asparagus stem 3.0 g/kg (AEAS-H). *P < 0.05 statistically significant difference from control.

The one-way ANOVA revealed a significant increase in the percent of time spent in open arms with AEAS treatment (1.5 and 3.0 g/kg) compared with the control (Figure 3). AEAS treatment increased the ratio between the number of entries in open arm and total entries, but these changes were not statistically significant. No differences were observed in total arm entries. As expected, DZP treatment yielded a significantly increased percent of open arm entries and time spent in open arms. 

### 3.2. Effect of AEAS in Vogel Test

**Figure 4:**
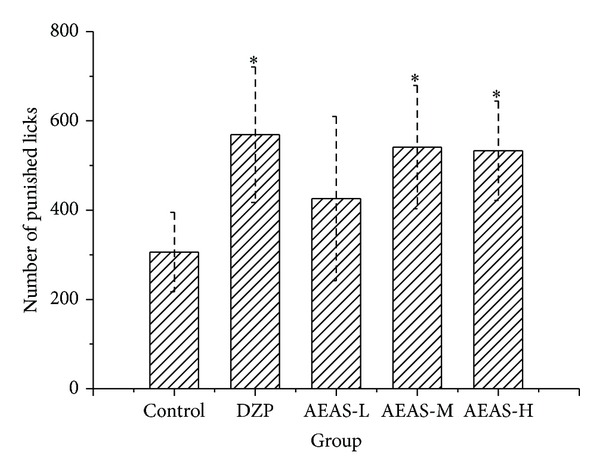
The effect of AEAS on the Vogel conflict test. The aqueous extract of asparagus stem (AEAS) and diazepam (DZP). The percent of open arm entries to the total number of arm entries (OE). The percent of time spent in the open arm to the total time in the open and close arms (OT). The dose of aqueous extract of asparagus stem 0.6 g/kg (AEAS-L). The dose of aqueous extract of asparagus stem 1.5 g/kg (AEAS-M). The dose of aqueous extract of asparagus stem 3.0 g/kg (AEAS-H). *P < 0.05 statistically significant difference from control.

To confirm the anxiolytic-like effect of AEAS, the Vogel conflict test was applied. In this experiment, one-way ANOVA revealed significant variance among the five groups. Doses of 1.5 and 3.0 g/kg significantly increased the number of punished licks compared with controls (Figure 4). As expected, DZP (3 mg/kg) significantly increased the number of punished licks.

### 3.3. Neurochemical Assay

**Figure 5:**
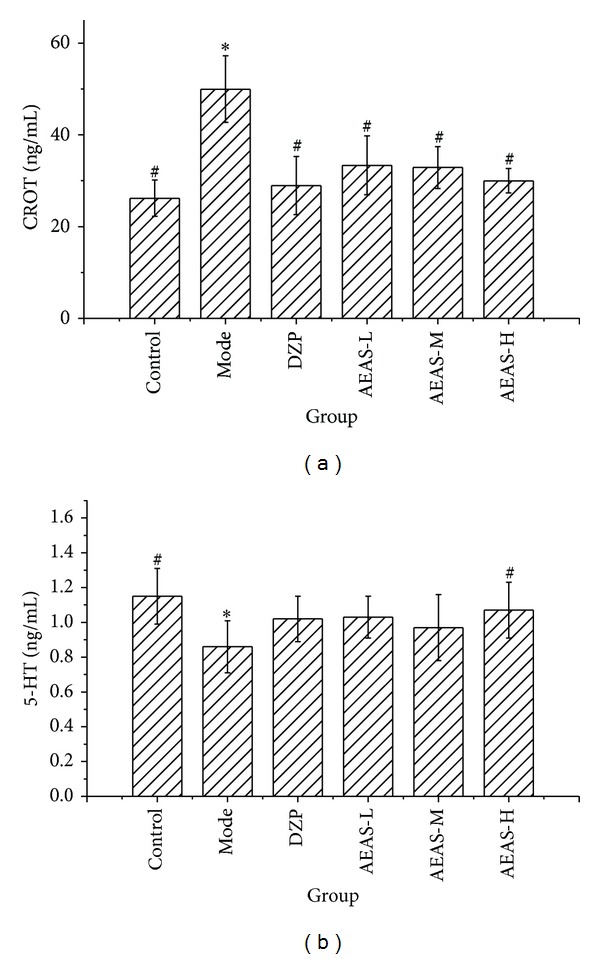
The serum biochemical levels in the Vogel conflict test ((a) for CROT and (b) for 5-HT). The aqueous extract of asparagus stem (AEAS) and diazepam (DZP). The percent of open arm entries to the total number of arm entries (OE). The percent of time spent in the open arm to the total time in the open and close arms (OT). The dose of aqueous extract of asparagus stem 0.6 g/kg (AEAS-l). The dose of aqueous extract of asparagus stem 1.5 g/kg (AEAS-m). The dose of aqueous extract of asparagus stem 3.0 g/kg (AEAS-h), Cortisol (CORT), and 5-Hydroxytryptophan (5-HT).*P < 0.05 statistically significant difference from control; ^#^ *P* < 0.05 statistically significant difference from model.

In the Vogel conflict test, the serum cortisol and epinephrine level increased significantly, compared with the control (Figure 5). AEAS treatment resulted in decreasing levels of serum cortisol. However, AEAS treatment produced an opposite effect on 5-HT, increasing the serum 5-HT level. Epinephrine levels decreased in AEAS- and DZP-treated groups, with no statistical significance, compared with the model group. We observed no significant changes in norepinephrine and dopamine levels between these groups.

The levels of epinephrine, 5-HT, norepinephrine, and dopamine in hippocampus homogenate showed no significant changes between groups (data not shown).

### 3.4. Repeated Doses Toxicity

All rats survived for the duration of the feeding trial. There were no notable changes in behavior, activity, posture, gait, or external appearance in any of the groups. There was no significant difference in average body weights or average daily food intakes between the rats in the control group and those in any of the treated groups during the study (for both sexes). All animals were subjected to complete necropsy. For the relative organ weight, statistical analysis result showed that there was no difference between groups (Table 4). The qualitative analyses of urobilinogen, urine glucose, urine acetone bodies, urobilinogen, urine protein, and urine nitrite were negative. The urine pH was in normal range in both of the control group and treated groups. As shown in Table 5, there was no significant adverse effect in any of the treated groups, compared with control group. Serum biochemistry results (Table 6) demonstrated that the parameters of biochemistry were within the normal range, and statistical analysis results showed that there was no difference between groups. In the histopathological study, there were no treatment-related changes in the main organs (for both sexes), whereas only sporadic and/or spontaneous lesions were observed similarly in the control and treated animals. The current study indicates no observable adverse effects on the rat reproductive system.

## 4. Discussion

The literature survey revealed that the steroidal saponins are the main biologically active constituents of Asparagus. In the previous pentobarbital-induced sleep test, linear regression analysis showed a correlation between asparagus saponin intake and increased sleeping time. Reports suggest that sarsasapogenin from *Anemarrhena asphodeloides* Bunge (Liliaceae) exile antidepressant activity by mediation of the central monoaminergic neurotransmitter systems [15, 16]. The content of steroidal saponins was quantified by determination of sarsasapogenin, as an index of active constituents of asparagus.

The present study sought to analyze the behavioral effects of AEAS in two animal models. The results showed that AEAS exhibited anxiolytic-like activity and mediated the secretion of cortisol and 5-HT. The neurochemical assay suggested that the anxiolytic effects of AEAS may be partly attributable to the modulation of neuronal and endocrine. The present findings may also provide important scientific evidence for the application and development of relaxation functional food to conquer the anxiety.

The elevated plus maze is one of the most widely used models and has been validated [18, 19], which uses the natural fear of rodents to avoid open and elevated places. When the animals were treated with the higher doses of AEAS (1.5 and 3.0 g/kg), anxiety-like behavior in the elevated plus maze was significantly attenuated, increasing the percent of time spent in open arms.

The Vogel test has become a standard for fast screening the potential anxiolytic properties of drugs. In this procedure, drinking behavior is punished by mild electrical shocks, leading to significantly reduced water consumption in deprived animals. To further validate these data, we tested the anxiolytic-like effects of these treatments in the VCT, which involves the suppression of punished responses. The behavioral suppression induced by shocks in the VCT is attenuated by anxiolytic drugs. The doses of 1.5 and 3 g/kg AEAS increased the number of punished licks (i.e., induced anxiolytic-like effects) to 177% and 174%. In the present study, diazepam was used as a positive control. As expected, it increased activity in the open arms of the elevated plus maze and number of punished licks in the VCT, confirming its anxiolytic actions.

As traditional medicine spreads extensively worldwide, edible herbs have become an increasingly important ingredient in functional foods and therapeutic drugs. In India, traditional folk medicine uses the roots of *Asparagus officinalis L*. to promote improvement in physical and mental health [11–13]. Component analysis showed that asparagus are rich in saponins from *Asparagus officinalis* (17.8%), aspartic acid (6.1%, accounting to one third of total amino acid), and gamma-amino butyric acid (1.0%). Aspartic acid is an amino acid that participates in hormone production and nervous system function. Specifically, aspartic acid contributes to fight fatigue and other symptoms related to stamina deficiency. The brain converts glutamine into glutamic acid and increases the amount of gamma-aminobutyric acid (GABA), which is needed to sustain proper brain function and mental activity [20, 21]. Naturally existing GABA in AEAS also participates importantly in relieving stress and exhibiting anxiolytic effect.

For the edible safety investigation, the acute toxicology study suggesting the maxim tolerant dose was 20.0 g/kg in this experiment condition. In the repeated dose oral toxicology study, the safety dose was 5.0 g/kg for 13 weeks.

In summary, the present study showed that AEAS exhibited strong anxiolytic-like effect and mediated the secretion of crotisol and 5-HT. These data demonstrated that acute stress (open elevated plus maze and punished licks) [22] elicits anxious behaviors and increases serum cortisol level. The AEAS treatment which decreases the secretion of crotisol suggested that the anxiolytic effect of AEAS may be partly attributable to the modulation of neuronal and endocrine. Therefore, AEAS is a promising candidate for the treatment of anxiety-like disorders in the alternative medicine.

## Figures and Tables

**Table 1:** Nutrient and chemical compositions of the AEAS.

No.	Compositions	Content	Unit
1	Carbohydrates	44.4	g/100 g
2	Proteins	24.5	g/100 g
3	Lipids	3.0	g/100 g
4	Calcium	3.84	g/100 g
5	Potassium	1.12	g/100 g
6	Magnesium	0.33	g/100 g
7	Sodium	0.28	g/100 g
8	Ferrum	0.06	g/100 g
9	Copper	0.19	g/100 g
10	Zinc	0.09	g/100 g
11	Vitamin E	0.17	mg/100 g
12	Vitamin B1	0.39	mg/100 g
13	Vitamin B2	0.89	mg/100 g
14	Selenium	0.14	mg/100 g
15	Saponins	17.80	g/100 g
16	Flavone	2.91	g/100 g
17	Polyphenol	5.93	g/100 g
18	Polysaccharide	11.21	g/100 g
19	GABA	0.82	g/100 g

AEAS: aqueous extract of asparagus stem; GABA: gamma-aminobutyric acid.

**Table 2:** Amino acid analyses of the AEAS.

No.	Amino acid	Content (g/100 g)
1	ASP	5.95
2	THR	0.29
3	SER	0.51
4	GLU	4.77
5	GLY	0.51
6	ALA	0.48
7	VAL	0.53
8	MET	0.07
9	ILE	0.16
10	LEU	0.41
11	TYR	0.23
12	PHE	0.15
13	LYS	0.29
14	HIS	0.11
15	ARG	0. 41
16	PRO	2.60
17	TRP	0.11
18	CYS	0.28

19	Total	17.86

AEAS: aqueous extract of asparagus stem.

**Table 3:** The spectrum data of the AEAS.

No.	UV (nm)	MS	MS/MS	Chemical name	Relative peak area
1	270 nm	595	[595]: 331 (100), 535 (19.65), 287 (8.19)	Kaempferol-3-O-*β*-rutinoside	1.00
2	270 nm	268	[268]: 136 (100)	7-hydroxy-4′-methoxyisoflavone	0.08
3	330, 260	611	[611]: 591 (100), 467 (38.97)	Rutin	0.65
4	340, 260	317	[317]: 298 (84), 198 (100)	Isorhamnetin	0.72
5	320, 280	301	[301]: 269 (100), 241 (99)	Quercetin	0.67
6					0.63
7					0.46
8					0.33

**Table 4:** Effect of AEAS repeated doses on relative organ weights (%) in rats.

Treatment	Dose (g/kg)	Gender	Heart	Liver	Spleen	Lung	Kidney	Brain	Adrenal	Thymus	Testis	Ovary
Control	—	♂	0.81 ± 0.06	4.29 ± 0.56	0.35 ± 0.06	0.75 ± 0.05	0.92 ± 0.11	1.05 ± 0.06	0.019 ± 0.002	0.04 ± 0.01	1.20 ± 0.11	
	—	♀	0.88 ± 0.06	3.74 ± 0.62	0.34 ± 0.04	0.71 ± 0.05	0.88 ± 0.11	0.95 ± 0.06	0.018 ± 0.003	0.03 ± 0.01		0.08 ± 0.01

AEAS	1.25	♂	0.82 ± 0.04	4.19 ± 0.49	0.35 ± 0.04	0.73 ± 0.04	0.92 ± 0.12	1.01 ± 0.04	0.021 ± 0.002	0.04 ± 0.01	1.19 ± 0.13	
	1.25	♀	0.92 ± 0.06	3.78 ± 0.58	0.34 ± 0.06	0.70 ± 0.04	0.86 ± 0.10	0.91 ± 0.05	0.019 ± 0.002	0.04 ± 0.01		0.08 ± 0.01
	2.5	♂	0.85 ± 0.04	4.01 ± 0.44	0.35 ± 0.04	0.75 ± 0.05	0.86 ± 0.08	1.05 ± 0.04	0.024 ± 0.003	0.04 ± 0.01	1.15 ± 0.11	
	2.5	♀	0.86 ± 0.04	3.69 ± 0.72	0.31 ± 0.05	0.72 ± 0.05	0.81 ± 0.09	0.95 ± 0.06	0.017 ± 0.003	0.03 ± 0.01		0.08 ± 0.01
	5.0	♂	0.86 ± 0.04	4.33 ± 0.53	0.36 ± 0.04	0.76 ± 0.04	0.91 ± 0.11	1.06 ± 0.04	0.022 ± 0.002	0.04 ± 0.01	1.26 ± 0.13	
	5.0	♀	0.86 ± 0.04	3.69 ± 0.41	0.33 ± 0.05	0.69 ± 0.04	0.84 ± 0.15	0.96 ± 0.04	0.018 ± 0.002	0.03 ± 0.01		0.08 ± 0.01

*: significantly different from the control group in the same gender at *P* < 0.05.

Results are expressed as mean ± SD, *n* = 10 animals/group.

**Table 5:** Effect of AEAS repeated doses on haemogram in rats.

Treatment	Dose (g/kg)	Gender	WBC (×10^9^/L)	RBC (×10^12^/L)	HGB (g/L)	PLT (×10^9^/L)	Lymph (%)	Neutrophil (%)	Monocyte (%)	Acidophil (%)	Basophils (%)
Control	—	♂	8.96 ± 2.16	7.11 ± 0.54	132 ± 10	326 ± 36	80.1 ± 3.3	14.3 ± 3.0	4.1 ± 0.6	0.9 ± 0.4	0.6 ± 0.3
	—	♀	7.54 ± 2.95	7.14 ± 0.51	133 ± 11	326 ± 36	79.6 ± 3.9	15.1 ± 4.5	4.1 ± 0.6	0.7 ± 0.3	0.5 ± 0.3

AEAS	1.25	♂	9.36 ± 3.13	7.03 ± 0.46	131 ± 10	336 ± 53	78.7 ± 3.6	15.9 ± 3.9	4.2 ± 0.6	0.6 ± 0.5	0.4 ± 0.3
	1.25	♀	6.98 ± 2.55	7.16 ± 0.52	130 ± 11	336 ± 53	79.5 ± 3.0	15.5 ± 3.2	3.6 ± 0.5	0.8 ± 0.4	0.6 ± 0.3
	2.5	♂	7.10 ± 1.88	7.04 ± 0.59	130 ± 12	370 ± 38	80.2 ± 3.6	14.9 ± 3.3	3.8 ± 0.6	0.6 ± 0.4	0.5 ± 0.4
	2.5	♀	7.71 ± 2.47	7.18 ± 0.53	131 ± 12	370 ± 38	80.6 ± 3.5	14.0 ± 3.7	4.0 ± 0.6	0.7 ± 0.4	0.5 ± 0.2
	5.0	♂	8.42 ± 2.47	7.15 ± 0.53	131 ± 14	342 ± 27	79.1 ± 3.6	15.4 ± 3.7	4.2 ± 0.6	0.8 ± 0.4	0.5 ± 0.3
	5.0	♀	7.65 ± 2.85	7.32 ± 0.57	131 ± 7	342 ± 27	78.6 ± 3.1	16.2 ± 3.4	4.1 ± 0.5	0.6 ± 0.3	0.5 ± 0.3

*: significantly different from the control group in the same gender at *P* < 0.05.

Results are expressed as mean ± SD, *n* = 10 animals/group.

The red blood cell count (RBC), hemoglobin concentration (HGB), lymphocyte concentration, neutrophil cells concentration, monocyte concentration, acidophil and basophils concentration, white blood cell count (WBC), and platelet count (PLT).

**Table 6:** Effect of AEAS repeated doses on biochemical parameters in rats.

Treatment	Dose (g/kg)	Gender	TP (g/L)	ALB (g/L)	T-CHO (mmol/L)	TG (mmol/L)	T-BIL (*μ*mol/L)	BUN (mmol/L)	CRE (*μ*mol/L)	ALT (IU/L)	AST (IU/L)	GLU (mmol/L)
Control	—	♂	70.5 ± 4.5	40.0 ± 3.4	1.76 ± 0.21	1.03 ± 0.15	1.56 ± 0.11	6.58 ± 0.36	38.5 ± 4.8	36.3 ± 3.7	128 ± 11	6.43 ± 0.34
	—	♀	70.6 ± 3.8	40.5 ± 3.1	1.65 ± 0.22	1.06 ± 0.12	1.51 ± 0.31	6.68 ± 0.28	37.2 ± 4.8	34.6 ± 4.2	126 ± 11	6.32 ± 0.23

AEAS	1.25	♂	70.6 ± 4.4	40.8 ± 3.4	1.70 ± 0.39	1.06 ± 0.13	1.60 ± 0.19	6.45 ± 0.25	37.3 ± 3.8	37.8 ± 4.4	130 ± 11	6.34 ± 0.30
	1.25	♀	71.4 ± 3.6	41.1 ± 3.2	1.66 ± 0.12	1.15 ± 0.13	1.58 ± 0.18	6.57 ± 0.24	38.5 ± 4.3	36.3 ± 4.4	123 ± 6	6.43 ± 0.30
	2.5	♂	71.4 ± 4.5	41.8 ± 3.3	1.82 ± 0.28	1.16 ± 0.14	1.62 ± 0.20	6.59 ± 0.26	35.6 ± 4.7	38.6 ± 4.4	123 ± 16	6.68 ± 0.27
	2.5	♀	69.1 ± 4.1	39.8 ± 3.7	1.73 ± 0.17	1.08 ± 0.14	1.69 ± 0.39	6.50 ± 0.15	36.5 ± 3.4	37.3 ± 5.2	130 ± 9	6.52 ± 0.33
	5.0	♂	69.7 ± 3.6	39.7 ± 3.2	1.65 ± 0.37	1.11 ± 0.18	1.55 ± 0.17	6.54 ± 0.26	39.0 ± 5.5	33.5 ± 6.9	133 ± 11	6.53 ± 0.32
	5.0	♀	68.4 ± 3.8	38.4 ± 4.2	1.69 ± 0.17	1.11 ± 0.18	1.57 ± 0.32	6.62 ± 0.18	36.5 ± 5.0	37.3 ± 4.8	127 ± 8	6.31 ± 0.35

*: significantly different from the control group in the same gender at *P* < 0.05.

Results are expressed as mean ± SD, *n* = 10 animals/group.

Total protein (TP), albumin (ALB), total cholesterol (T-CHO), triglycerides (TG), total bilirubin (T-BIL), blood urea nitrogen (BUN), creatinine (CRE), aspartate aminotransferase (AST), alanine aminotransferase (ALT), and blood sugar level (GLU).
